# The “maintainer's” perspective on maintaining regular physical activity: theory development using a constructivist grounded theory approach

**DOI:** 10.3389/fspor.2026.1869609

**Published:** 2026-09-30

**Authors:** Livia Kellerhals, Claudio R. Nigg

**Affiliations:** Department of Health Science, Institute of Sport Science, University of Bern, Bern, Switzerland

**Keywords:** long-term, maintenance, physical activity, qualitative, theory

## Abstract

**Background:**

The factors leading to physical activity maintenance are not well-understood. This study investigated such factors among individuals who adhere to the WHO physical activity guidelines for five years or longer, exploring perspectives, motives and facilitators of long-term physical activity maintenance.

**Methods:**

A constructivist grounded theory study was conducted to explore the phenomenon through the maintainers themselves (*n* = 10) via in-depth semi-structured interviews.

**Results:**

Coded segments (*n* = 1,127) were sorted into 21 themes and summarized into six broad categories: three main categories: 1) enjoyment; 2) adaptability; 3) well-being, the fundamental factor of 4) priority, as well as two supporting categories: 5) organization, and 6) community. The model presents the interconnectedness of these factors.

**Conclusion:**

This study aligns and expands the existing literature on physical activity maintenance, emphasizing the importance of prioritization as foundational for adaptability, enjoyment, and well-being. The findings suggest that a dynamic approach is crucial for physical activity maintenance.

## 1 Introduction

Recent data shows that 27.5 percent of the global population is insufficiently physically active (1). The current World Health Organization (WHO) physical activity (PA) guidelines recommend: 150–300 min of moderate-intensity aerobic PA or 75–150 min of vigorous-intensity aerobic PA. In addition, it is recommended to do strength training twice a week as well as to actively reduce sitting time (2). Meeting the WHO PA guidelines protects against non-communicable diseases such as type II diabetes mellitus or coronary heart disease and reduces the risk of premature death by 20–30 percent, among other benefits (2). These benefits are achieved through moderate- and vigorous-intensity aerobic PA as well as muscle-strengthening activities. Additionally, not only do muscle strengthening exercises enhance musculoskeletal and mental health, but they also help in decreasing visceral fat (3). Although some benefits can already be achieved below the recommended levels of aerobic PA and muscle-strengthening activity, higher levels are associated with greater health benefits (4). Furthermore, increased PA has been shown to effectively reduce healthcare costs (5).

PA and, more generally, behavior change interventions can successfully increase PA levels (6, 7). However, maintaining these behavioral changes over time remains challenging, with many interventions reporting substantial dropout or relapse rates within six months after completion (6). Consequently, evidence on the long-term maintenance of behavior change remains scarce. Although numerous behavior change theories describe factors influencing behavior, relatively few explicitly address the maintenance of behavior (8). Theories that do address maintenance in some form include the transtheoretical model [TTM; (9)], social cognitive theory [SCT; (10)], the Physical Activity Maintenance (PAM) Theory (11), Rothman's theory of maintenance (12), and habit theory (13).

General behavior-change theories typically conceptualize maintenance as one component of a broader change process. The TTM defines maintenance as a distinct stage characterized by sustained behavior, increasing self-efficacy, management of temptations, and relapse prevention (9). SCT explains maintenance through reciprocal interactions among personal, behavioral, and environmental factors, particularly self-efficacy, outcome expectations, goals, self-regulation, and social or environmental support (10). Habit theory adds that repeated performance in stable contexts can create cue–behavior associations, allowing behavior to be initiated more automatically and with less reliance on conscious motivation (13).

Maintenance-specific theories propose more explicitly that sustaining a behavior requires processes that differ partly from those involved in its initiation. The PAM Theory (11) identifies maintenance specific goal attainment, satisfaction and commitment; self-motivation and expectations; and barrier and relapse self-efficacy as key determinants of sustained PA, while also emphasizing supportive environments and life stress. Similarly, Rothman et al. (12) propose that initiation is driven primarily by expected outcomes, whereas maintenance depends more strongly on satisfaction with outcomes actually experienced and may increasingly involve automatic processes. Thus, although these theories share constructs such as self-efficacy, goals, environmental support, and self-regulation, the PAM theory and Rothman's framework focus more specifically on the distinctive demands of maintenance, including managing relapse, adapting to stress and barriers, experiencing sufficient rewards, and developing more automatic behavioral control.

In addition to the relative paucity of maintenance in theories of behavior, the scarcity of the evidence is also partly due to the absence of an agreed-upon operational definition of behavioral maintenance, influencing whether and how the topic is studied (14). The Health Maintenance Consortium (HMC) conceptually defines behavioral maintenance as continued behavior demonstrated during a specific period after an intervention, meeting a certain threshold believed to enhance well-being or health (15). The definition includes periods of behavior interruption or lapses and does not categorize them as maintenance failures (15). The operationalized definition applied in this study aligns with the summarization of most operational PA maintenance definitions described by Dunton et al. (16). By adopting the WHO guidelines, the required threshold and frequency of PA are defined, while the 5-year cut-off operationalizes the duration and consistency of PA maintenance (16). Consequently, the operationalized definition applied in this study contains adhering to the WHO guidelines or surpass the level of aerobic PA and strength training. Furthermore, this definition uses a time cut-off of five years, as suggested by the TTM for the termination stage, which is rarely reached but implements the consistency aspect (9).

Recent publications have aimed at providing overviews and reaching a consensus on maintenance, such as those by (8, 16–18). Determinants of behavioral maintenance mentioned in these and other theories include self-efficacy, -determination, -perception, and -regulation, which strongly depend on a person's psychological and physical resources, as well as environmental, social and diverse motivational aspects (8). Although these theoretical explanations have advanced understanding of behavioral maintenance, they have been derived primarily from existing evidence and researcher-led conceptualizations and may not fully capture the dynamic, context-dependent, and fluctuating nature of long-term physical activity maintenance. Quantitative research can establish the prevalence, strength, and temporal patterns of associations among predefined variables, whereas qualitative research can complement these findings by examining how and why maintenance occurs and by identifying meanings, experiences, contextual influences, and unanticipated mechanisms that may not be represented in existing theories or standardized measures (19, 20). Moreover, the perspectives of the target population—particularly individuals who have successfully maintained physical activity over time—remain largely absent from this literature. As key stakeholders with direct lived experience, long-term maintainers may identify practical strategies, adaptive processes, critical turning points, and interactions among personal, social, and environmental influences that are difficult to anticipate from theory alone. Involving target users can therefore enhance the contextual relevance and ecological validity of theoretical models and support the development of interventions that are more acceptable, feasible, and responsive to users' needs and everyday circumstances (21, 22). Therefore, these theoretical explanations and possible predictions of behavioral maintenance ought to be informed by and tested in a variety of settings and population groups of long-term maintainers to promote further development of theories concerning PAM (11, 17, 18).

For the further development of these theoretical explanations, it is crucial to understand the way that various health determinants are experienced and understood by the target population—the people whose health are sought to be understood or promoted. Previous studies on health behavior have primarily relied on quantitative methods (23), and the exploration of qualitative data acquired directly from individuals with a consistent engagement in PA has been limited. Thus, a qualitative approach will capture the unique perspectives and valuable insights of these individuals who have been successful at maintaining long-term PA.

Therefore, this study aimed to explore the factors and underlying mechanisms of long-term PA maintenance from the perspective of individuals who had successfully maintained the WHO PA recommendations for at least five years.

The results will be cross-referenced with existing theories and potentially present a new conceptualization.

## 2 Methods

### 2.1 Study design

A qualitative study design using a constructivist grounded theory approach (24) was applied to show the complex phenomenon of PAM from the perspective of the maintainers. The constructivist grounded theory approach helps reveal the hidden assumptions behind familiar concepts and focuses on generating new theories through inductive analysis of the data gathered from participants rather than from pre-existing theoretical frameworks (24).

### 2.2 Participants and sampling

Following ethical approval by the ethics committee of the Faculty of Human Sciences, University of Bern (Nr.2023-04-09), all procedures were carried out in accordance with institutional guidelines, relevant national laws, and the Declaration of Helsinki. Informed consent was obtained in writing from all participants prior to data collection. Participant recruitment began with an online survey. This consisted of a validated short German Physical Activity Questionnaire (short-GPAQ) (25), the short Muscle-Strengthening Exercise Questionnaire (MSEQ) (2) translated into German by the first author, and demographic questions. In accordance with current reporting guidelines, participants were asked to indicate their sex assigned at birth (male/female). Gender identity was not assessed in this study; therefore results refer to biological sex rather than gender identity. A criterion-based, purposive sampling strategy was applied, with the aim of maximizing variation (26) and ensuring that the participants' PA levels conformed to the operational definition of maintenance applied in the study. Of the 78 individuals who completed the survey, three participants were chosen based on their demographic characteristics and activity patterns to attempt the acquisition of potentially comprehensive and diverse data. These participants were subsequently contacted either via email or phone, depending on the contact information provided in the questionnaire.

The inclusion and exclusion criteria, as well as the recruitment strategy, are presented in Table 1. These criteria and strategies were carefully devised to identify a diverse set of individuals that all meet the criteria of maintainers, as outlined above.

**Table 1:** Recruitment plan.

Recruitment plan	
Inclusion criteria	Adults, 18–64 years old, fulfilling the WHO guidelines Bull et al. (2) for at least 5 years
Exclusion criteria	Being a professional athlete
Recruiting through	Social media, mailing list, sports clubs and other associations, flyers in grocery stores, fitness centers, gyms and snowball sampling
Selection criteria	Different forms of activities, all gender, age, living environment, educational level, years being physically active

In accordance with the inductive design of the constructivist grounded theory approach the recruitment of participants continued after the beginning of the interview process. Out of this process three more interviews were conducted and enabled the researchers to make informed decisions regarding additional recruitment, as they initiated the transcription and open-coding processes in parallel. Thus, after conducting six interviews, theoretical sampling was applied to search for younger adults, who were not health professionals, as by this point certain commonalities emerged between the older participants and those working in healthcare. In total, eleven interviews were conducted, but only the data of ten participants were included, with diverse geographical backgrounds and different activity levels, as described in Table 2. One participant was excluded as her activity profile changed during the recruitment phase, and thus no longer met the defined PA maintenance definition and did not intend on re-adopting the PA maintenance as defined, thus no further information was collected from this participant. Furthermore, the participants displayed a wide range of educational backgrounds, spanning from completed apprenticeships to master's degrees.

**Table 2:** Participant characteristics.

	Activity/week mod + vig	Strength training/week	Age	Sex	Living environment	Educational level	Time active in years
ID 43	150 Min	2 × 15 Min	32	F	City	BSc (HP)	8
ID 71	400 Min	3 × 120 Min	22	F	Agglomeration	Ma (HP)	13
ID 75	240 Min	2 × 60 Min	61	M	Rural	MSc (HP)	30
ID 77	305 Min	4 × 60 Min	46	F	Rural	BSc	27
ID 105	360 Min	2 × 60 Min	33	M	Rural	App	10
ID 108	2,400 Min	2 × 60 Min	33	M	Rural	App	5
ID 138	270 Min	2 × 30 Min	52	F	Rural	Diploma (HP)	35
ID 169	345 Min	3 × 60 Min	64	F	Agglomeration	Diploma	10
ID 183	840 Min	3 × 90 Min	24	M	City	App	19
ID 184	360 Min	3 × 15 Min	21	F	City	Ma	8
MEAN (SD)	567 (670) Min	2.6 × 57 Min	39 (19)	60% F	50% Rural	30% University	17 (11)

Description 1: Sex (biological) F, female; M, male; Activity/week mod + vig = through short-GPAQ self-reported amount of activity/week (work, exercise, leisure time) moderate (mod) and vigorous (vig) added up; strength training/week = through MSEQ-short self-reported strength training sessions per week; Educational Level BSc, bachelor of science; MSc, master of science; Ma, matura/secondary school; App, apprenticeship; Diploma, professional school, HP, health professional.

Their activity profile ranged from individual sports such as gym workout, brisk walking, cycling, or high intensity interval training, to team sports like volleyball, gymnastics, and other voluntary sports clubs' activities. The duration of their PAM varied between five and 35 years.

### 2.3 Data collection

The first author, a trained physiotherapist, conducted all the interviews. The interview guide was developed with a set of main questions that were asked in each interview, with follow-up questions and probes as needed. The development of the questions included a group discussion with students and instructors within the discipline, followed by a review of the questions with an expert in the field. Finally, the interview questions were pilot tested with two individuals, the list of the final questions are provided in Table 3.

**Table 3:** Interview questions.

Category	Question(s)
Opening Question	You have already given me your details on exercise in the survey, why don't you tell me again in what form you are physically active during a normal week, including strength training? 1.1 And (if not already mentioned) how often and how long do you engage in your regular physical activity?
Main questions	2. What does regular physical activity mean to you personally?
	3. And what does it mean for your everyday life? For your environment?
	4. What would you say is the main reason why you are physically active?
	5. How have you managed to maintain this active lifestyle for so long?
Additional questions	6. When and how did your current active lifestyle begin?
	7. Do you have the impression of having factors in your life that act as barriers to maintaining physical activity?
Closing question	8. Is there anything you would like to add that you haven’t mentioned, or I haven't asked about?

The qualitative data collection was conducted through in-depth, semi-structured interviews. The interviews' duration was between 30 and 50 min, and they were all conducted in person, were in Swiss German, and were audio recorded. Some of the interviews were conducted at the university, at participants' workplace or in public spaces such as coffee shops or restaurants. These different locations were chosen and agreed upon to decrease time burden, inconvenience, and ensuring the highest possible comfort level for the participants. Confidentiality during the interviews was ensured through using made-up names during the interviews.

All participants gave their written and oral, informed consent to the audio recording, as well as to the use and quoting of their interviews in a pseudonymized form. The consent form they signed informed them about the research question and the general aim of the research.

The questions were adapted as necessary throughout the interview process. Thus, the more data were gathered, the more specification questions and probes specific to gain more in-depth information on the question asked were used in the interviews. The researcher took field notes during the interviews, including an own commentary, as well as critical reflection on possible biases directly after the interviews. The data collection took place over a period of three months, with continuous transcription of the recordings. After the third interview, parallel line-by-line coding on the collected data was started.

### 2.4 Data analysis

Data were analyzed according to the constructivist grounded theory approach including an initial line-by-line coding process followed by focused coding (24). To ensure credibility, one interview was coded by both authors independently, revealing a high level of agreement (>90%) of the codes and themes identified. Subsequent coding was carried out by the primary author.

The data were transcribed and coded using the MAXQDA2022 Software (VERBI GmbH Berlin, Germany, Version 22.8.0). Some codes and themes were repeated and by the eighth interview, theoretical saturation concerning the topic was achieved, which was confirmed after conducting two additional interviews, further emphasizing the importance of the identified themes through in-depth analysis of the diverse participants' experiences. Thus, sampling was ultimately completed with ten long-term active participants. The line-by-line coding process yielded 234 codes and subcodes, encompassing over 1,000 coded segments. Constant comparison was utilized during open coding and conducting further interviews to merge the collected data and compare the different participants to each other (24).

Through the continuous focused coding process a total of 33 codes and subcodes emerged. Two coders collaborated to establish 17 themes and 4 subthemes out of these codes. Throughout the process, detailed memos were written to capture ideas, compare codes, and address any uncertainties and biases. The codes underwent further refinement through merging and deletion, while free writing and diagramming techniques were employed to elevate the codes to a more abstract level. Through an ongoing iterative process of transitioning from raw data to identifying themes, comparisons were made among the themes, revealing interconnections that were then visually organized and distilled into 6 overarching factors. Importance was determined by a combination of the number of times themes/factors were mentioned (at least 5 times) and by how many out of the 10 participants (at least 3) mentioned them. Finally, mindmapping and interpretation discussion within the study team was done until consensus was reached. This process (24) culminated in the emergence of a conceptual framework, which is detailed in the subsequent findings.

Once the themes were established and the model was developed, they were returned to the participants with a request for feedback and confirmation if they reflected their experience. Three of the ten participants provided feedback that was positive overall, and no feedback was received from the remaining seven. The only feedback that was critical of the model concerned the lack of visibility for the factor of habit, which was featured as a subtheme of *adaptability.* This will be further elaborated in the section on findings and the discussion.

## 3 Results

Of the 1,127 coded segments, 21 themes were deemed relevant to the research question. These 21 themes were organized into six broad categories for PAM: 1) Well-being, 2) Adaptability, 3) Enjoyment, 4) Priority, 5) Organization and 6) Community.

These were subsequently divided into the three core factors for PAM, *well-being*, *adaptability,* and *enjoyment;* the two supporting factors of *organization* and *community;* and the fundamental factor of *priority*.

While *priority* and *organization* were mentioned more often, see the bracketed numbers in Figure 1, *adaptability* and *enjoyment* were rated as more important for the maintenance process. This is reinforced by the broad support from the data gathered from maintainers themselves, as every single person in the sample named *adaptability* and *enjoyment* as crucial to their PAM. Accordingly, the relative strengths of the different factors' influence on PAM is illustrated by the width of the arrows in Figure 1.

**Figure 1:**
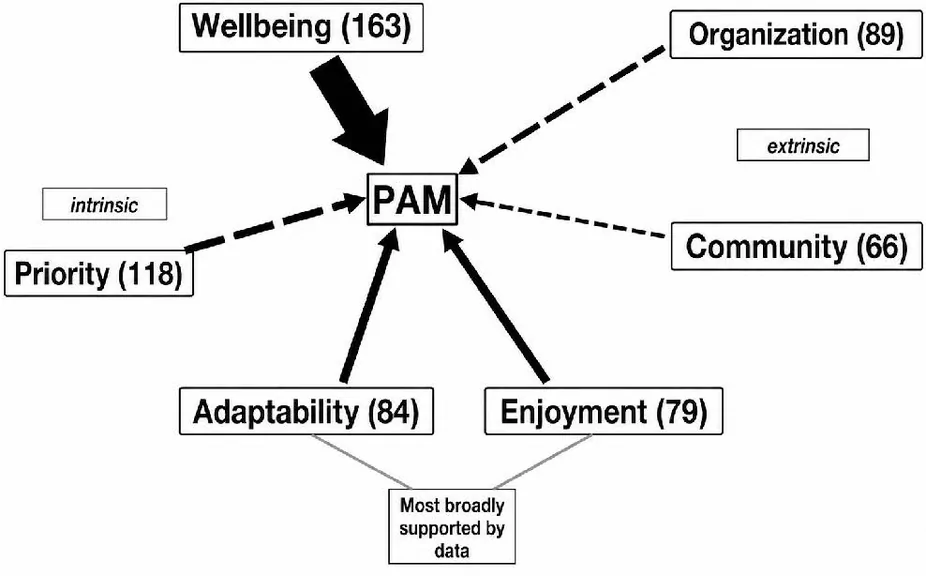
The relative strength of the different factors’ influences on PAM, according to the weighting and to the frequency of their mentions in the interviews.

All factors, including the supporting elements, are interconnected with one another. This intricate web of connections forms a cohesive system, where each component both supports and influences the others. While some categories may have a one-way influence as outlined in Figure 2, they ultimately loop back into the larger interconnected system, thus rendering all categories interdependent.

**Figure 2:**
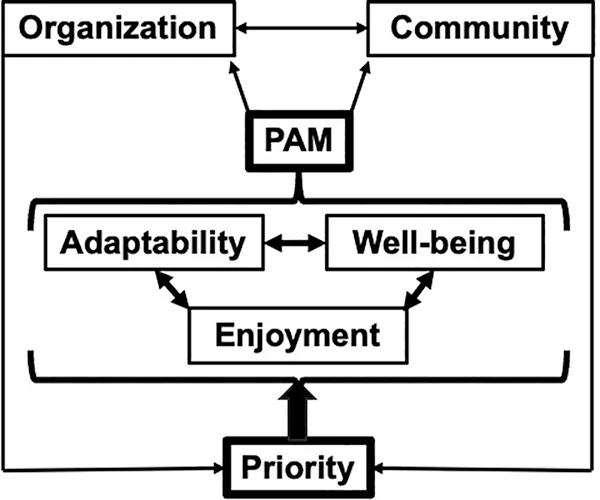
PAM model based on the qualitative input of physical activity maintainers.

The model shows ascending arrows starting from *priority* and moving upwards, in the direction of PAM, through its three core factors. As a consequence of the maintenance process, *community* and *organization* emerge and develop, forming a reciprocal link back through the factor of *priority*. As a result, the cycle of PAM is continuously maintained. The subsequent sections provide an in-depth description of the factors and their interconnections within the model, as shown in Figure 2.

### 3.1 Description and relations of the factors

#### 3.1.1 Initiation by the priority factor

*Priority* encompasses various variables influencing participants' PAM, including compulsion (*n* = 41)^1^, prioritization (*n* = 35), goals and success (*n* = 21), increased productivity (*n* = 11), renunciation (*n* = 10), perseverance (*n* = 4), and self-motivation (*n* = 6). Despite the diversity of these variables, they all play a crucial role in starting and sustaining PA engagement, and they all stem from a self-regulatory approach.

Participants feel guilty when unable to adhere to exercise routines, highlighting compulsion and guilt as driving factors for PAM. For instance, ID 138 describes the perceived pressure to incorporate movement into every day:

“And there simply has to be some sort of movement in my day, at least once, and that stresses me out a little sometimes, of course. Because I simply have the feeling that I should be doing it now. (ID 138, Pos. 27)”

This illustrates how the sense of obligation towards PA can become a source of stress when daily routines are disrupted, reflecting the compulsive dimension of prioritization described by several participants.

Prioritization also comes with actively renouncing other activities in favor of PA, demonstrating a commitment to maintaining routines. ID 184 describes:

“For sport. So it's true, even though I have a full program now with my studies, I don't have time at the weekend (because of volleyball matches), I just work 10 h a day from Monday to Friday and then I'm done. So yes, I don't think I would ever cancel sport to do something else. (ID 184, Pos. 15)”

Participants' overall PA goals are aligned with their various life goals, such as keeping their weight stable, aging well or getting stronger for specific tasks, motivating them to continue being active. Success in achieving parts of these goals serves again as a motivator to maintain their training. For example, ID 169 describes:

“So now with the strength training of the legs also with cycling, that just gets easier… (ID 169, Pos. 83)”

The variables within the factor *priority* of increased productivity and self-motivation are linked by the participants to PA, enhancing daily activities. For instance, ID 169 describes how PA directly enhances her daily productivity:

“Variety *laughs*. Yes, very pleasant, so I think I get things done faster afterwards, when I *laughs*. I think the housework would take me longer if I just had a whole morning at home, but then I go to training and afterwards everything just goes a bit faster and easier. So yes, a welcome change of pace. (ID 169, Pos. 21)”

Self-motivation is described as an intrinsic drive that stems from within. It enables participants to maintain their activity levels effortlessly, as it is not reliant on external factors, but on their internal motivation instead, which is described as naturally high by some of the participants.

Taken together, these variables underline the high level of importance that maintainers assign to PA in their lives.

As described above, *priority* is a fundamental factor in PAM. Nevertheless, it is not sufficient on its own. As illustrated in Figure 2, the importance of this factor lies in its role in starting and reinforcing the triangular cycle of the key factors. It is essential for PA and serves as a fundamental starting point, indicating that PA must have a certain level of importance and *priority* in one's life for it to be maintained.

The participants' intrinsic motivation aligns closely with habit formation, as they find that sticking to their PA routines becomes second nature due to their inherent drive and commitment, showcasing the connection to *adaptability*. The variables of prioritization and renunciation influence *adaptability*, as the priorities can change. Through the variable of compulsion, *adaptability* can be influenced negatively, but as described above, compulsion seems to be an effective means of maintaining activities.

Well-being is further influenced by the variables falling under the factor of *priority,* such as increased productivity, resilience, and perseverance. These traits are highly consequential for happiness, satisfaction and with that for overall *well-being*. Furthermore, *enjoyment* is also connected to *priority*. Having fun increases the *priority* of PA in an individual's life and reinforces the willingness to plan, adapt and to perform the activity effectively.

### 3.2 Maintenance and triangular reciprocity

#### 3.2.1 Enjoyment

This core factor to PAM contains the variables of enjoyment (*n* = 53) and of variation (*n* = 26). All participants reported experiencing genuine enjoyment stemming from specific sports and activities they engage in regularly. While most participants mentioned that some form of PA has always been part of their lives, they also reported having had to experiment and explore different activities before discovering what truly resonated with them, ultimately leading them to commit to it. *Enjoyment* emerged as a key motivating factor for PAM, highlighted by all participants, as for instance by ID 77:

“And yes, having joy, joy, joy in movement, joy in what you do, and yes that's sometimes something you have to find out somehow. (ID 77, Pos. 61)”

Furthermore, the variable of variation is mentioned as important, as participants reported that even once they had found their preferred sport or activity, they continue seeking out new experiences and exploring different forms of movement. They embrace variety within their routines to keep PA engaging and exciting, emphasizing the need for diverse activities to maintain motivation. For instance, ID 75 describes actively seeking out alternatives to avoid monotony:

“And yesterday for example I had the feeling, no, I don't want to do my standard tour on the road again, and then I took the mountain bike and went through the forest, and I liked it because it was a change. (ID 75, Pos. 85)”

Similarly, ID 184 describes her approach to strength training as driven by spontaneous preference rather than fixed routine:

“…strength training I just do otherwise, at home. I used to go to the gym, not so much anymore *both laugh*, but just on Monday or Tuesday or on the weekend, just random stuff that I somehow do, so not any specific topic or anything in particular, just whatever I feel like doing at the time. (ID 184, Pos. 3)”

#### 3.2.2 Well-being

*Well-being* is the second core factor in the data, with three main pillars identified: mental balance and relaxation (*n* = 68), overall health (primarily physical, *n* = 48), and general well-being (*n* = 47). Mental balance and relaxation were highlighted as important benefits of PA, serving as a motivation for individuals to maintain their routines. ID 108 for example, describes finding it in running:

“It's just the perfect way to get rid of thoughts or simply to air out a bit. (ID 108, Pos. 41)”

The second pillar, overall health, was particularly prominent among participants over the age of 40 and those working in healthcare, who emphasized the importance of physical health for motivating regular exercise. These participants described receiving increased feedback from their bodies over time, prompting them to adapt their routines accordingly. ID 138, for instance, describes this process:

“I had always been all about high-intensity sports, I did aerobics, I went jogging, I went walking, I was in the gymnastics club, and then over time I noticed a bit that it wasn't only doing me good, I started getting tension everywhere and back pain and so on. And then I realized, hey now the time has come, well you don't get any younger, now I think I need to focus a bit more on flexibility. And relax a bit more, and then I chose yoga. (ID 138, Pos. 31)”

General well-being was described as positive feelings associated with being physically active, including happiness, satisfaction, and fulfillment. These aspects have a strong influence on participants' commitment to PAM, as shown in Figure 1.

#### 3.2.3 Adaptability

*Adaptability* emerged as the third core factor for participants' PAM, and it contains two main variables. One is flexibility and experience (*n* = 52), and second is habit (*n* = 25). Additionally, a third variable, the need to move, was mentioned by a smaller number of participants (*n* = 7).

Participants demonstrated significant adaptability over 5–35 years of PAM, adjusting their routines in response to changes in their life circumstances, such as changing workplaces. They showed flexibility in finding alternative ways of staying active and developed habits through long-term engagement in PA. ID 71 states:

“…otherwise I always have a way, so work has never been an obstacle either. So when I was in my remote internship, I just made sure that I could go to training there. I was doing an internship at location 9 and couldn’t commute to my regular gym, so I just made sure that I could go to gymnastics there, and that was really great. To see something new for once. (ID 71, Pos. 31)”

Some participants developed a perceived need to move regularly due to their habitual exercise routines, emphasizing the integral role of movement in their daily lives. As described by ID 77:

“…so it rarely takes a lot of effort, but in most cases it's just something that really extremely belongs in my everyday life, like drinking coffee, or whatever. (ID 77, Pos. 53)”

The data shows that even when life is relatively stable, there are always small adjustments to be made, and over time there are always major life changes. Interestingly, amidst all these changes, PA served as an anchor of stability in their lives. Adaptability from the perspective of the maintainers refers to both adapting PA choices and adapting one's life around one's PA habits.

#### 3.2.4 Interconnections between the key factors

This section further clarifies the relations between the three maintenance factors, as seen at the center of Figure 2, in the triangle of reciprocity.

The relationship of *enjoyment* to *well-being* is evident in the maintainers' experience of joy through movement and in the concomitant sense of happiness and satisfaction (*well-being*). Like ID 105 states:

“I enjoy pushing myself to my physical limits. Because I actually find that afterwards it's such a satisfying feeling. A feeling of well-being, of happiness; “hey, you've done something”. It fulfills you. That's why I think that if I find something that I like doing, then I want to do it for as long as possible. (ID 105, Pos. 53)”

Arguably the factor of *enjoyment* could be summarized as well under the factor of *well-being*. In the context of PAM, it is still separated as *enjoyment* is a crucial motivating factor and should not be lost through mixing it with other factors. The same applies for *adaptability* as it could be seen as a self-regulatory skill and as such belongs to the factor *priority*.

As seen in Figure 2, *adaptability* and *enjoyment* are directly linked. This connection is driven by the variable of variation, a subcode of *enjoyment* (see Section 3.2.2): the ability to introduce variety into PA enhances the overall experience. Maintainers actively seek out new ways to keep their activities engaging and enjoyable, even after years of consistent participation. This pursuit of variation not only adds fun but also contributes to *adaptability* by increasing the variability of their experiences and their readiness to tackle diverse situations.

Flexibility is an essential component in the maintainers' physical activities, particularly when dealing with injuries or illnesses, necessitating adaptations based on their current health conditions. This dynamic illustrates the interconnectedness of *adaptability* with *well-being*. As described above, maintainers such as ID 71 actively sought out alternative training opportunities when their circumstances changed, demonstrating their capacity to adjust their routines in response to shifting conditions while preserving their overall *well-being*. Conversely, *well-being* also influences *enjoyment*, as participants remarked that engaging in PA with pain diminishes the fun factor and can be quite bothersome. As described above, these three key factors are the drivers of the maintenance process. Upon achieving PAM, two supportive factors emerge to enable its continuation: *Organization* and *community*.

This interconnectedness of the factors was not solely an analytical finding but was also directly articulated by the participants themselves. When reflecting on what enables long-term maintenance, some maintainers spontaneously described a holistic perspective in which PA is embedded within a broader lifestyle framework encompassing multiple health-related behaviors. ID 183 illustrates this perception:

“Yes, generally the lifestyle… For example smoking or such things, alcohol, otherwise just being more conscious, engaging with it, going through life, eating healthy, variety I think is also important. Yes, you can't really, yes it is actually an overall picture that has to be somewhat right so that you can achieve that, I think. (ID 183, Pos. 87)”

This perspective reflects how maintainers perceive PA not as an isolated behavior but as one component within a coherent system of health-related choices—including nutrition, substance avoidance, and a conscious approach to daily life—that must align for long-term maintenance to succeed. The emphasis on the need for an “overall picture” and for variety directly mirrors the reciprocal relationships among the factors identified in the model, particularly the interplay between *well-being*, *adaptability*, and *enjoyment*.

### 3.3 Supporting factors and consequences of PAM

#### 3.3.1 Organization

Organizational aspects (*n* = 54) and the structure of voluntary sports clubs (*n* = 34) are key components under the umbrella of *organization*. Coaching, counseling, and guided training sessions (*n* = 4) also fall within this category.

Effective planning and organization throughout the week, such as minimizing travel time between commitments and combining exercises for efficiency, play a significant role in PAM. Participants emphasize the importance of structured weeks and regular working hours for consistent activity. Environmental aspects, like surroundings, influence participants' PAM. The maintainers prioritize workout locations for convenience and time efficiency. ID 183 even describes that one of the criteria to choose his new workplace was the location:

“Mhm, yes, I've been working here since September, so it was a logical criterion for me to keep distances short. We train twice at location 15, and I can just walk down from here and get there quickly. (ID 183, Pos. 17)”

Involvement in voluntary sports clubs from a young age was described by several participants as fostering personal development through gradually taking on responsibilities and leading training sessions. ID 71 describes this progression:

“…yes, and then you get small tasks, like responsibilities, where you have to organize something here or there, and that ties you in as well, because you then feel like you are important in the club and you can give something back. (ID 71, Pos. 23)”

The organizational framework of voluntary sports clubs, along with the social factors embedded within them, encourages commitment and accountability. While these structures were not the primary focus of this study—as not all participants were members of sports clubs—they emerged as a relevant organizational factor for those who were involved and may warrant further investigation. The same organizational principles apply to other forms of coached and guided training sessions, which similarly increase accountability and reduce barriers to activity.

#### 3.3.2 Community

The factor of *community* encompasses three primary variables: social support (*n* = 28), social interactions and connections within sports (*n* = 25), and a sense of belonging (*n* = 13). Participants receive support from partners and sports teams, which motivates them to stay active. This support is particularly crucial after a long workday, when fatigue sets in. Interestingly, the participants often found themselves in the role of motivators within their social circles. Friendships formed through sports and long-term engagement in sports clubs or classes foster strong connections and motivate continued activity. ID 71 states:

“…but on the other hand, it's just fun because I see (in the sports club) colleagues that I've known for 14 years, that I've known for ages, and it just feels really good. (ID 71, Pos. 11).”

This feeling of belonging not only supports their PAM but becomes an element in their identity. Some participants view themselves as parts of an active population, distinguishing themselves from the inactive segment of the population. This expanded sense of belonging enhances their commitment to regular PA.

#### 3.3.3 Interaction of the three supporting factors

Notably, these supporting factors exhibit strong interdependence. The factors of *community* and *organization* are tightly intertwined, as most maintainers take part in regular, planned activities (*organization*) and build part of their *community* through this.

Conversely, individuals may also plan their activities around their sports partners, as seen in activities like climbing or jogging. For example, ID 43 describes that she coordinates her bouldering with her friends:

“That they like… I can also say that I can actually go bouldering like every evening, it doesn't really matter, I coordinate with my friends. And I don't have the problem of irregular working hours that keeps you from going to the same group, which always takes place the same evening, so I don't have that problem. (ID 43, Pos. 27)”

Both *community* and *organization* contribute to the PAM cycle by influencing the factor of *priority* and thus connecting with the key factors. The effect of *community* on *priority* can be seen, for example, in the fact that meeting friends for sports increases the willingness to sacrifice other things and thus reinforces the prioritization of training. A supportive social environment can also enhance perseverance and help in reaching activity goals. The higher the importance placed on PA in life, the more it supports motivation to plan and organize time, which shows the mutual connection between *organization* and *priority*. On the other hand, the more the structural environment and for instance the equipment are organized, the easier it becomes to prioritize PA.

*Organization* is closely intertwined with *adaptability*, as individuals need to organize and plan their activities with some flexibility. For example, some maintainers have exercise equipment at home that they can use in case the weather conditions are bad. ID 75 states:

“I still have an exercise bike at home, so even if it's really “ugly” outside or snowy or something like that, then I can also use the exercise bike, but I really find that the most tedious thing, I really only do it out of health awareness and so that I simply don't lose my form. (ID 75, Pos. 85)”

Or they plan their life and activities around seasonal requirements of their sports (e.g., the volleyball season).

Figure 2 shows the link between the factors of *community* and *well-being*, and through that also to *enjoyment*. A supportive community or positive interactions in sports enhance individuals' enjoyment, thereby boosting their motivation to engage in PA. These interactions also contribute to their overall well-being, as a strong social network significantly impacts one's sense of *well-being*.

## 4 Discussion

### 4.1 Summary of the main findings

The aim of this research project was to contribute to the complex and understudied subject of the maintenance process in PA behavior. To achieve this objective, this study collected data from individuals, who have consistently adhered to the WHO's PA guidelines for over five years (2). The model presented here was developed from the analysis of interviews with ten physically active individuals with different backgrounds, surroundings, ages, and activities. The findings from this study provide a qualitative data-driven model for an in-depth understanding of PAM in a diverse population, with the aim of bridging the existing knowledge gap in PAM research, with a particular focus on a holistic approach (18), emphasizing the interrelationships among the various influencing factors.

The factors of *well-being, adaptability, enjoyment, priority, organization,* and *community* are intricately interconnected and form a cohesive system where each component influences and supports the others.

*Priority* serves as a starting point for the core factors, emphasizing the importance of PA in one's life. *Enjoyment* is a fundamental prerequisite for sustained engagement in PA, affording the maintainers mental and physical benefits (*well-being*) that are motivating to retain them in their lives, and *adaptability* plays a crucial role in facilitating the maintenance of a consistent level of PA over time. The two consequential factors to further support PAM, *organization*, and *community*, emerge. *Organization* encompasses aspects such as effective planning, and facilitates PAM. *Community*, on the other hand, includes the social environment of the maintainers, which is seen as supportive in motivating individuals to stay active. Overall, the interconnectedness of *organization* and *community* enhances PAM by supporting *priority*. Thus, the factors of the PAM cycle reinforce each other, and the cycle is continuously maintained.

### 4.2 Situating the reciprocal physical activity maintenance theory

Most of the vast PA literature is focused on behavior change, PA adoption and promotion (18). Certain features of the model presented here reflect to some extent the predominant theories and hypotheses that emerged from the reviews.

At the center of the model the three main factors *enjoyment, adaptability* and *well-being* are closely interrelated and form a triangle of reciprocal support and reinforcement. These factors align to some extent with the five main themes outlined by Kwasnicka et al. (8). The factor of *enjoyment* in the model coincides with the maintenance motives presented in the systematic review. *Enjoyment* also emerged from the overview as a key driver of adherence to PA. Interestingly, the model presented in this study highlights the search for variation that maintainers actively employ to keep activities engaging and interesting. This process of seeking diversity plays a crucial role in sustaining excitement about PA by experimenting with and discarding various forms of exercise. This is supported by facets of affective-reflexive theory (ART) in searching for positive affective responses (or enjoyment) associated with different physical activities (27). This is also in agreement with the description of PAM in Nigg et al. (11) as a continuous active process and in addition, it could be due to an elevated fitness status, which requires new and higher loads to trigger relevant physiological responses (17). This also reflects a process of selecting an activity by weighing the pros and cons, as captured in the concept of the decisional balance in the TTM (9). This process was quite important and is still part of participants' PAM process. Comparing the model presented here with the TTM is difficult, as the TTM is a stage model, and the maintenance process alone is a multifaceted, complex phenomenon (28). The current data point to the continuous, cyclical nature of PAM.

The second main motive described by Kwasnicka et al. (8) is satisfaction with the behavior, which in this model is part of the second main factor, *well-being*. The participants described feelings of happiness, satisfaction and even fulfillment due to PA and to adherence to a certain PA routine. Especially the feeling after exercising is described as satisfactory: several maintainers reported that the feeling of significant exertion during exercise leads to a sense of fulfillment after finishing the exercise. This aspect of the factor of *well-being* appears to be supported by ART and the affective responses triggered by the intensity of exercise coupled with its corresponding physiological reactions (27). The sense of fulfillment derived from adherence to PA is further enhanced by the sub-theme of identity, a concept underscored by Kwasnicka et al. (8) as a primary motivator. In the current model, identity is subsumed under the factors of *community,* as “sense of belonging”, and under *priority*, as “self-motivation” which serves as an important catalyst and sustaining engagement in the triangle of reciprocity between the core factors of the model in the present study, and thereby reinforcing PAM. This emphasis on *priority* reinforces PAM and ensures its continuation by solidifying one's identity as an active and sportive individual, which in turn is intensified through the continued demonstration of this behavior over time. This evolution of identity through habitual engagement is also explained in the model of Rhodes (29) and is recognized as one of the main maintenance motives by both Kwasnicka et al. (8) and Rhodes & Sui (18).

The factor of *well-being* aligns partially with the third theme outlined in the review of Kwasnicka et al. (8): “Resources”. Resources and the constraints posed by limited physical and psychological assets and the elevated risk of lapses that ensues are discussed extensively in other literature as well (11, 16). Lapses and recoveries are even used as possible indicators to operationalize PAM (14). The data provided by the maintainers themselves indicate that once maintenance is achieved, the demand for resources and adherence requirements does not need to remain consistently high to sustain PA levels. In fact, PA is intentionally utilized to enhance both physical and psychological resources and to effectively manage situations and periods of heightened stress. This strategic use of PA as a coping mechanism is particularly relevant in mitigating the impact of life stressors, which have been identified as potential triggers for relapses and as factors that impede PAM (11). Interestingly, the data showed that PA was deliberately maintained by the participants in intense and challenging periods and used much like an anchor. This again underscores that once the maintaining stage is reached, PAM becomes a resource that is consciously used. The utilization of PA as a resource was also named as a strategy for recovery following injuries, moving to a different city, as well as the various burdens and limitations imposed by restrictions during the Covid-19 pandemic. This demonstrates a high level of flexibility in behavior maintenance, which can be seen as flexible habits (17), as discussed further in the next section.

The factor of *adaptability* in the model contains the theme of habit, which overlaps with the third theme in the systematic review of Kwasnicka et al. (8). Habit formation through regular long-term engagement in PA is important from the maintainers' perspective. They identify specific cues, such as the signal of every Wednesday after work, which prompts them to cycle straight to their training sessions. These stimuli are described as being part of the automatic processes of behavior, particularly in the context of dietary behavior (12). The extent to which health behavior, such as PA, can become habitual is controversial, and habit appears to primarily influence its initiation rather than its execution (13). This aligns with the cues for going to training sessions described by the maintainers above. The execution itself can be viewed as an active process that necessitates continual adaptation to the individuals’ circumstances and current states, while at the same time requiring the ability to resist and renounce other interests, as outlined under the factor of *priority*. This element of renouncing or minimizing negative cues has been shown to be effective in changing addictive behaviors (30).

As mentioned above, the habitual initiation of PA developed by the maintainers needs to be combined with the additional element of significant flexibility to ensure long-term adherence. As the results presented here indicate, the continuous adjustment of PA routines makes their longevity more likely. This is consistent with the above description of PAM as the continued, active application of psychosocial strategies (11), and possibly also a constant shifting between automatic and controlled processes (16). This ability to shift is essential both for behavior maintenance and for resuming the behavior following a lapse (16). The broad range of experiences gained through their varied routines enhances their adaptability to diverse situations and life stressors and improves their ability to navigate challenges effectively.

A unique feature of this model is that, instead of limiting itself solely to habit, it considers a broad spectrum of diverse experiences as a crucial factor in maintenance. This broad spectrum is nurtured and sustained by the variety of different forms of PA that is deliberately kept by maintainers as part of their routines, to preserve the element of fun in their physical activity routine, as described under the factor *enjoyment* above.

The factor *adaptability* within the model also encompasses part of the self-regulation and self-efficacy aspect outlined in the TTM (9) and in the PAM theory (11). The adaptability demonstrated by maintainers underscores the significance of self-regulation processes, which align with the second theme “self-regulation” identified by Kwasnicka et al. (8).

These key factors within the model exhibit a strong interplay and mutual influence, characterized by a triangular reciprocity and direct interaction. This dynamic differs from the proposed interrelationships among the five theoretical themes in the systematic review by Kwasnicka et al. (8), which tends towards a more unidirectional model structure.

The three supporting factors described in the model *priority, organization* and *community* are interrelated as well but can be distinguished through their nature, *priority* being a starting point and reinforced through the cyclical nature of maintenance, whereas *organization* and *community* are more consequential factors developed through PAM. In addition this addresses to some extent the issue of the temporal sequence of factors, a research gap identified in behavior maintenance studies 18). This differentiation aligns with existing literature. *Community* and *organizationa*l factors are viewed as environmental and social influences, while *priority* predominantly pertains to self-regulation and self-efficacy processes.

The factor *community* contains the supportive environment described in several studies, a big part of the participants described being active already since a young age or having had active role models within their families. Some but not all were engaged in a sport during childhood. Which supports that individuals are more likely to follow the example given by people they trust and feel connected to (10). This early imprint by the family is not mentioned in the model as it is not a continuous maintaining factor and as that does not apply to the research question. But according to the ART the affective experiences, on the base of learning, leave traces on the memory, which would support the familial imprint as important to how PA is experienced and with that influencing *priority* as a factor within the maintenance process (27).

The supportive environment described by Nigg et al. (11) shows a high agreement with the factors *organization* and *community* described here as well as within the fifth theme “environmental and social influences” by Kwasnicka et al. (8). Interestingly the social support and community aspect was described by the participants as being relatively small because of the high self-motivation that they developed through the long-term activity, which again reflects the difference between initiation and long-term maintenance.

The information presented here, along with its contextualization, should be interpreted within the framework of long-term PAM spanning from 5 to 35 years. This extended duration underscores a remarkable level of adherence, revealing diverse and different facets of PAM.

### 4.3 Practical implications

Although the model presented in this study is a new framework for understanding PAM and the interrelationships between the main motives, which needs further testing, especially when it comes to its applicability, it contains potential implications for future PAM program design. PAM interventions should have a focus on offering a wide variety of physical activities, to promote a wide range of experiences, but also to foster enjoyment as excitement rises with variety. Programs should address the aspect of adaptability by guiding or teaching the planning itself of PA routines. The benefits of a dynamic and flexible approach for behavior maintenance should be understood by the participants. However, replication, confirmation and validation of these results is required prior to solidifying practical implications.

### 4.4 Limitations

A common limitation of qualitative studies is that the researcher plays a crucial role in collecting and analyzing data and inevitably brings her/his own beliefs, values and experiences along, potentially influencing the processes of data collection and analysis (24). Efforts were made to reflect prior knowledge and bias about the topic, the beliefs and own experiences were noted and discussed with other researchers in the field, to minimize their effect on the data collection and analysis. The bias that may have exerted the strongest influence was that the interviews were conducted by a health professional, a fact that was disclosed to the maintainers. Consequently, many participants were recruited through associated health professional colleagues, who share a particular interest in the subject matter. This recruitment method may have introduced a bias into the data, potentially inflating the perceived health benefits as part of the primary motivators. Another limitation of the qualitative research process applied in this study is that it captures the human experience through the story of the participants (26). The data collection was restricted to the researcher's questions during interviews, without any additional methodological approaches, and the data relied solely on self-reported activities. Although the questions were designed to be open-ended to capture a wide range of experiences, some aspects of PAM may have been missed.

These common limitations of qualitative research were minimized by reporting and relying closely on the consolidated criteria for reporting qualitative research (COREQ) (31).

Generalizability is limited as all participants of this study self-identified as able-bodied and Caucasian. It is further limited as everyone recruited lives in Switzerland. Switzerland is one of the most physical active populations in the western world, with notable density of voluntary sports clubs, outdoor recreational opportunities and infrastructures like gymnasiums even in rural areas (32). The recruitment strategy and purposeful sampling tried to minimize the homogeneity of the participants but as in grounded theory the data emerges from the information of the participant this model may be limited in its generalizability.

A difficulty encountered within this study was that the WHO guidelines provide only a broad definition of strength training, making it challenging for participants to determine if, for example, their yoga class is sufficient and for researchers to decide when and how to include specific participants. Consequently, determining the appropriate classification for participants, for example for which their main sport is climbing, can be challenging. The MESQ-Short (2) showed to be a good support to make sure that the included participants engage in some sort of muscle-strengthening activity.

## 5 Conclusion

By studying individuals who have successfully adopted and maintained long-term PA, an important contribution to understanding PAM was made. The approach of investigating health behaviors among people who have successfully incorporated PAM could be further used in the research, as PAM is still lacking clear definitions and requiring studies that last longer than 6 months. The model presented in this study aligns with existing PAM literature, while also offering a unique perspective on the foundational aspect of *priority* through the interplay and mutual influence of the three core factors *adaptability*, *enjoyment,* and *well-being*. *Enjoyment* and *adaptability*, and with that experiences and variation are highlighted and indicate their importance when it comes to promote and prioritize PAM. The findings suggest that a dynamic approach and flexibility within behavior maintenance are essential for long-term adherence to PA. To some extent the research gap of the interrelations and timing of important factors for PAM is thematized with this study. Future research is needed to enhance the understanding of PAM and to further test the applicability of the model presented and explore its implications for designing effective PA interventions.

## Data Availability

The raw data supporting the conclusions of this article will be made available by the authors, without undue reservation.
